# Effectiveness of respiratory muscle training on pulmonary function recovery in patients with spinal cord injury: a systematic review and meta-analysis

**DOI:** 10.7717/peerj.20373

**Published:** 2025-11-28

**Authors:** Shuqi Yao, Haozhe Guo, Fuhai Ma, Aiping Chi

**Affiliations:** 1 School of Physical Education, Shaanxi Normal University, Shaanxi, China; 2 School of Physical Education, Shanxi Datong University, Shanxi, China; 3 School of Physical Education, Qinghai Minzu University, Qinghai, China

**Keywords:** Respiratory muscle training, Spinal cord injury, Pulmonary function recovery, Systematic review, Meta-analysis

## Abstract

**Objective:**

This study conducted a thorough review and meta-analysis to examine how respiratory muscle training (RMT) affects lung function recovery in individuals with spinal cord injury (SCI).

**Methods:**

We conducted a systematic review of Randomized Controlled Trials (RCTs) examining the effects of RMT on lung function in patients with SCI. The search included databases such as PubMed, Embase, The Cochrane Library, Scopus, and Web of Science up to October 2025. The experimental group received RMT as the main intervention, while the control group received either no treatment, a placebo, or conventional rehabilitation. Outcome measures included Forced Expiratory Volume in the first second (FEV_1_), Forced Vital Capacity (FVC), Maximum Inspiratory Pressure (MIP), Maximum Expiratory Pressure (MEP), Peak Expiratory Flow (PEF), Minute Ventilation Volume (MVV), Total Lung Capacity (TLC), Inspiratory Capacity (IC), and Vital Capacity (VC). Two reviewers independently screened, extracted data, and assessed bias. Meta-analysis was conducted using RevMan 5.3 software, and the quality of included studies was evaluated using the Cochrane bias risk assessment tool and the Physical Therapy Evidence Database scale. The reporting of this study followed the PRISMA guidelines and was registered with PROSPERO (ID: CRD42024627736).

**Results:**

In this meta-analysis, 25 RCTs were included, comprising a total of 679 patients. The meta-analysis showed that compared with conventional rehabilitation, respiratory muscle training significantly improved FEV_1_ (*p* < 0.0001), FVC (*p* = 0.0001), MIP (*p* < 0.00001), MEP (*p* = 0.0004), PEF (*p* < 0.00001), MVV (*p* < 0.0001), TLC (*p* = 0.05), VC (*p* = 0.04), and their differences were statistically significant. However, IC (*p* = 0.40) was not statistically significant. Subgroup analyses showed that resistive training and surface electromyography biofeedback training were effective for improving FEV_1_ and FVC, while threshold training significantly improved MVV.

**Conclusion:**

This meta-analysis provides strong evidence that RMT is an effective intervention for enhancing respiratory muscle strength and key parameters of pulmonary function in individuals with SCI. Further research with robust methodologies and extensive sample sizes is needed to validate this finding.

## Introduction

Spinal cord injury (SCI) is a serious condition that affects the structure and function of the spinal cord, caused by factors like trauma, inflammation, and tumors (Choi et al., 2021). There are more than 20.6 million people worldwide living with SCI, with around 900,000 new cases reported annually (Mohammadi, Villeneuve & Smith, 2023). SCI can result in severe motor, sensory, and respiratory issues, significantly impacting patients’ quality of life and social interactions, and placing a burden on families and society (Courtine & Sofroniew, 2019; Lorach et al., 2023; Skinnider et al., 2024). Respiratory problems are a leading cause of death in SCI patients, as they can lead to pulmonary complications and respiratory failure (Brown et al., 2006; Berlowitz, Wadsworth & Ross, 2016). Research has found a negative correlation between the level of injury and respiratory parameters (Moreno et al., 2012). Individuals with high cervical or thoracic injuries often experience more profound respiratory dysfunction compared to those with paraplegia, due to greater impairment of respiratory muscle innervation (Shin et al., 2019). Respiratory problems are a leading cause of death in SCI patients, as they can lead to pulmonary complications and respiratory failure (Brown et al., 2006). Therefore, it is crucial to promptly rehabilitate respiratory function and provide appropriate respiratory support to help individuals with SCI recover.

In respiratory rehabilitation practice, conventional approaches include postural drainage, manually assisted coughing techniques, respiratory muscle endurance training, and mechanical ventilation support (Fabero-Garrido et al., 2024). These methods aim to facilitate secretion clearance, maintain airway patency, improve ventilation efficiency, and prevent complications such as atelectasis and pneumonia (Bott et al., 2009; Turcios, 2020). However, the effectiveness of traditional methods is often limited by factors such as patient compliance, requirements for operational expertise, and individual variability in treatment response (Zheng et al., 2025).

Respiratory muscle training (RMT), a non-invasive intervention, is beneficial for improving respiratory muscle function and reducing respiratory complications in individuals with SCI (Kumru et al., 2023). This intervention includes exercises that focus on both inspiratory and expiratory muscles to enhance strength, endurance, and coordination (Jackson & Groomes, 1994; Arora et al., 2012). Studies have demonstrated the effectiveness of RMT for individuals with SCI, showing improvements in lung function during early recovery and the prevention of a significant decline in lung function and cough capacity (Boswell-Ruys et al., 2020). Common outcome measures include lung volumes and flows, such as Maximum Inspiratory Pressure (MIP), Maximum Expiratory Pressure (MEP), Forced Expiratory Volume in the first second (FEV_1_), Peak Expiratory Flow (PEF), Minute Ventilation Volume (MVV), and Forced Vital Capacity (FVC) (Berlowitz & Tamplin, 2013; Tamplin & Berlowitz, 2014).

The efficacy of RMT in improving pulmonary function in patients with SCI is still debated (Templeman & Roberts, 2020; Ramli et al., 2023), despite its recognized positive potential. Current research has produced conflicting findings, with varying study quality and a lack of systematic evaluation. Research has shown that RMT demonstrates potential for improving respiratory function even in chronic SCI patients (Rutchik et al., 1998; West et al., 2014; Sankari et al., 2024). This study aims to conduct a comprehensive assessment of the available evidence through systematic evaluation and meta-analysis to determine the specific effect of RMT on lung function recovery in SCI patients.

The study aimed to investigate the impact of RMT on respiratory parameters in SCI patients. The goal was to analyze the effects of RMT on various indices including FEV_1_, FVC, MIP, MEP, PEF, MVV, Total Lung Capacity (TLC), Inspiratory Capacity (IC), and Vital Capacity (VC) in SCI patients. The study aimed to determine if RMT is more effective in improving respiratory function in SCI patients compared to conventional rehabilitation. The aim was to provide stronger evidence for clinical practice, assist healthcare professionals in making evidence-based decisions, and guide future research efforts. The findings of this study will be significant for clinical medicine and sports science by supporting the use of RMT in SCI patient rehabilitation and offering insights for enhancing athletic performance and health through respiratory training.

## Methods

The study was reported following the guidelines of the Preferred Reporting Items for Systematic Reviews and Meta-Analyses (PRISMA) (Moher et al., 2009). The protocol for this review is registered in the PROSPERO systematic review database (CRD42024627736).

### Search strategy

Two researchers conducted separate searches on various databases including PubMed, Embase, The Cochrane Library, Scopus, and Web of Science for studies on RMT in the treatment of spinal cord injuries. They utilized keyword searches, free-word searches, and logical operations with conjunctions. The search covered studies from the establishment of the databases up to October 2025. Terms used in the search included SCI, Traumatic Myelopathies, Spinal Cord Traumas, Post-Traumatic Myelopathy, Spinal Cord Lacerations, Spinal Cord Transections, Spinal Cord Contusions, Spinal Cord Lacerations, and Respiratory Muscle Training. Additionally, terms like Randomized Controlled Trial, Randomized, Placebo, Drug Therapy, and Randomly were included in the search.

### Study selection

The research was conducted in English and released in English peer-reviewed journals. The selection criteria for the study were developed around the PICOS (Participants, Intervention, Comparator, Outcome, and Study Design) framework as follows:

Inclusion criteria: (a) Participants: Adults (18 years of age or older) diagnosed with SCI; (b) Intervention: RMT as primary intervention; (c) Comparator: Control group receiving placebo, conventional rehabilitation, or no intervention; (d) Outcomes: Pulmonary function outcomes such as FEV_1_, FVC, MIP, MEP, PEF, MVV, TLC, IC, and VC; (e) Study design: RCT.

Exclusion criteria: (a) Studies not using RMT methodology in the intervention; (b) Studies using the same data as included studies; (c) Incomplete raw data or inability to extract outcome metrics; (d) Non-randomised controlled trials; (e) Unavailability of full text of the literature.

### Data extraction and synthesis

Two researchers (Shuqi Yao and Haozhe Guo) independently reviewed literature titles and abstracts to exclude irrelevant articles based on specified criteria. Following a comprehensive analysis of the full text, the literature was reevaluated and chosen for inclusion, with data extraction encompassing general information, study design, sample size, intervention method, assessment time, and outcome indicators. Any disagreements during the search process were first resolved through discussion between the two researchers. If consensus could not be reached, Aiping Chi served as the arbiter to make the final decision.

Two researchers used the Cochrane Collaboration tool to assess the risk of bias (Higgins et al., 2011) and Physiotherapy Evidence Database (PEDro) scale for quality assessment (Maher et al., 2003; Albanese et al., 2020). The PEDro scale had a total of 10 points (11 entries, with the first entry not counting towards the total). Scores were categorized as follows: <4 for low-quality literature, 4–5 for average-quality literature, 6–8 for higher-quality literature, and 9–10 for high-quality literature (Maher et al., 2003). The evaluation results were reviewed by two staff members, and any discrepancies were resolved through discussion within the research team.

### Statistical analysis

Statistical analysis was performed using RevMan 5.3 software, with data entry undergoing multiple checks to prevent errors. All outcome indicators in this study were continuous variables. Measurement data were analyzed using mean difference (MD) or standardized mean difference (SMD) with its 95% CI, while count data were analyzed using relative risk (RR) with its 95% CI for efficacy analysis. The X^2^ test was used to assess heterogeneity among the results of the included studies. If there was no statistical heterogeneity among the studies (*p* > 0.1, *I*^2^ < 50%), the fixed-effect model was used for meta-analysis. In cases of statistical heterogeneity among studies (*p* ≤ 0.1, *I*^2^ ≥ 50%), an investigation was conducted to identify the source of heterogeneity, and subgroup analyses were performed for factors that may be responsible for the heterogeneity. If clinical heterogeneity was eliminated, either a random-effects model was used for analysis or descriptive analyses were conducted. A *p*-value below 0.05 was considered statistically significant. Publication bias was assessed by creating a funnel plot.

## Results

### Literature screening process

**Figure 1:**
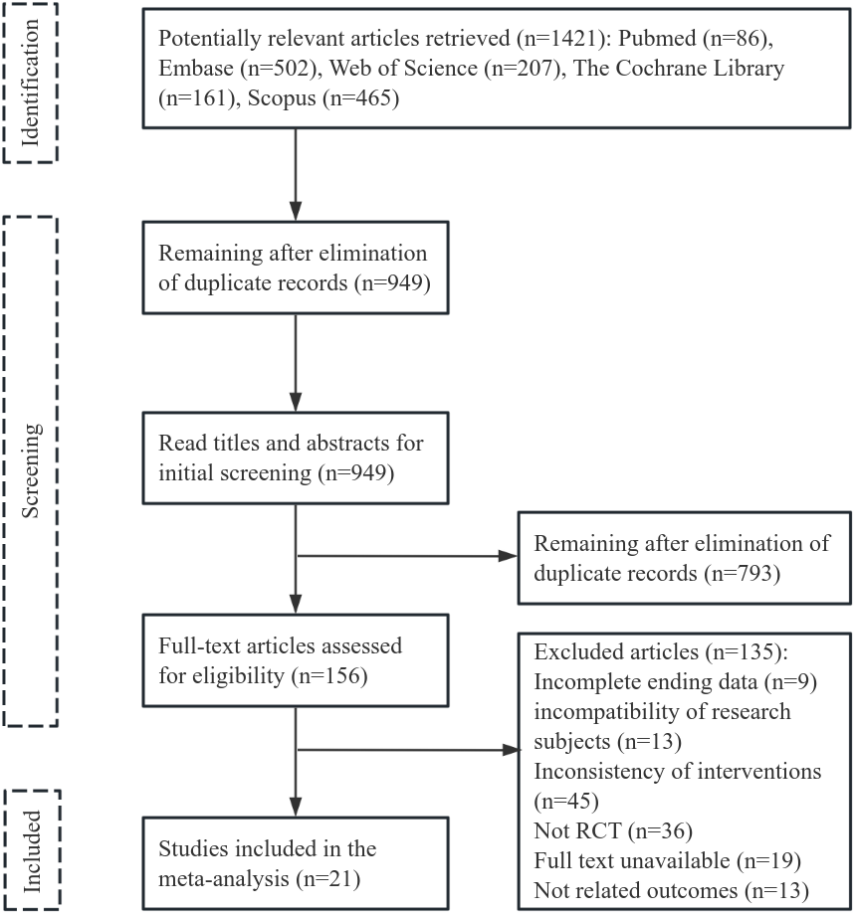
Literature selection process.

Initially, 1,421 articles were reviewed, 472 duplicates were deleted, and 949 articles were excluded after title and abstract assessment. The remaining 156 articles were read in full for potential inclusion. Ultimately, 21 articles were selected for the meta-analysis, with a combined sample size of 679 cases. The flowchart of literature screening can be seen in Fig. 1.

### Study characteristics

Supplemental Files A outlines the features of the trials included in the meta-analysis. The study involved 339 cases in the treatment group and 340 cases in the control group, all of whom were SCI patients who underwent RMT as an intervention. The specific interventions for RMT are presented in Supplemental Files B.

### Quality assessment

**Figure 2:**
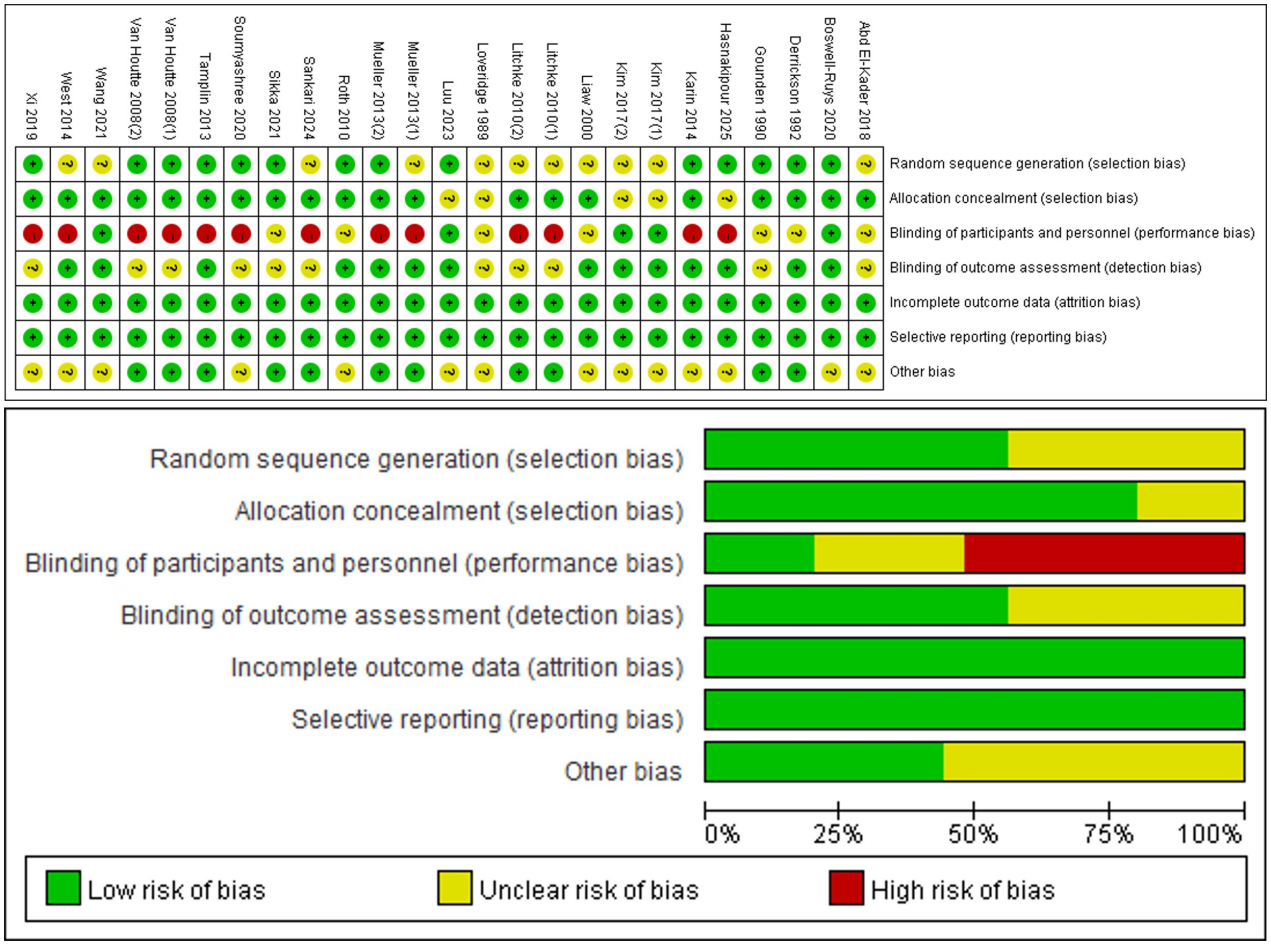
Risk of bias assessment. Note. Liaw et al., 2000; Roth et al., 2010; Mueller, Hopman & Perret, 2013; Tamplin et al., 2013; West et al., 2014; Postma et al., 2014; Kim et al., 2017; Abd El-Kader, 2018; Xi et al., 2019; Boswell-Ruys et al., 2020; Gitanjali et al., 2021; Sankari et al., 2024; Hasnakipour et al., 2025; Derrickson et al., 1992; Gounden, 1990; Litchke et al., 2008; Loveridge, Badour & Dubo, 1989; Luu et al., 2023; Soumyashree & Kaur, 2020; Van Houtte et al., 2008; Wang et al., 2021.

All 25 RCTs (Loveridge, Badour & Dubo, 1989; Gounden, 1990; Derrickson et al., 1992; Liaw et al., 2000; Van Houtte et al., 2008; Roth et al., 2010; Mueller, Hopman & Perret, 2013; Tamplin et al., 2013; West et al., 2014; Postma et al., 2014; Kim et al., 2017; Abd El-Kader, 2018; Xi et al., 2019; Soumyashree & Kaur, 2020; Boswell-Ruys et al., 2020; Gitanjali et al., 2021; Wang et al., 2021; Luu et al., 2023; Sankari et al., 2024; Hasnakipour et al., 2025) included in the analysis reported on the baseline situation of the patients. Twleve RCTs (Gounden, 1990; Derrickson et al., 1992; Van Houtte et al., 2008; Roth et al., 2010; Tamplin et al., 2013; Postma et al., 2014; Xi et al., 2019; Soumyashree & Kaur, 2020; Boswell-Ruys et al., 2020; Gitanjali et al., 2021; Luu et al., 2023; Hasnakipour et al., 2025) mentioned the use of a specific randomization method such as computerized randomly generated numbers or a random number table. Five RCTs (Boswell-Ruys et al., 2020; Postma et al., 2014; Soumyashree & Kaur, 2020; Tamplin et al., 2013; West et al., 2014) described the specific procedure used for allocation concealment. Two RCTs (West et al., 2014; Postma et al., 2014) were multicenter RCTs. Nine RCTs (Van Houtte et al., 2008; Mueller, Hopman & Perret, 2013; Tamplin et al., 2013; West et al., 2014; Postma et al., 2014; Xi et al., 2019; Soumyashree & Kaur, 2020; Sankari et al., 2024; Litchke et al., 2008) were single-blind. Four RCTs (Boswell-Ruys et al., 2020; Kim et al., 2017; Luu et al., 2023; Wang et al., 2021) were double-blind. Eleven literature outcome indicator assessors were blinded (Boswell-Ruys et al., 2020; Derrickson et al., 1992; Kim et al., 2017; Liaw et al., 2000; Luu et al., 2023; Mueller, Hopman & Perret, 2013; Postma et al., 2014; Roth et al., 2010; Tamplin et al., 2013; Wang et al., 2021; West et al., 2014). All articles provided a description of missing outcome data or reasons for missing data were described. The risk of bias in allocation and study results can be seen in Fig. 2.

The highest score among the included literature was 10 points, (Boswell-Ruys et al., 2020) while the lowest score was 5 points. (Gounden, 1990; Abd El-Kader, 2018; Gitanjali et al., 2021) Overall, the quality of the included literature was high, with scores concentrated in the range of 5–10 points (Supplemental Files C). PEDro scores of 9–10 points were classified as high-quality literature, while scores of 6–8 points were classified as higher-quality literature, and scores of 4–5 points were classified as average quality literature. Scores less than 4 points were classified as low-quality literature. One article in the current literature had a high-quality with a PEDro score of 10 points, (Boswell-Ruys et al., 2020) primarily due to its use of an intention-to-treat analysis. Seventeen articles were considered higher-quality literature (Loveridge, Badour & Dubo, 1989; Liaw et al., 2000; Van Houtte et al., 2008; Roth et al., 2010; Mueller, Hopman & Perret, 2013; Tamplin et al., 2013; West et al., 2014; Postma et al., 2014; Kim et al., 2017; Xi et al., 2019; Soumyashree & Kaur, 2020; Wang et al., 2021; Luu et al., 2023; Sankari et al., 2024; Hasnakipour et al., 2025; Litchke et al., 2008), with deficiencies in allocation concealment and intention-to-treat analyses.Three articles were average-quality literature (Gounden, 1990; Abd El-Kader, 2018; Gitanjali et al., 2021). primarily due to insufficient information on follow-up procedures and lack of blinding during the intervention.

### Synthesis of the results

#### FEV_1_

**Figure 3:**
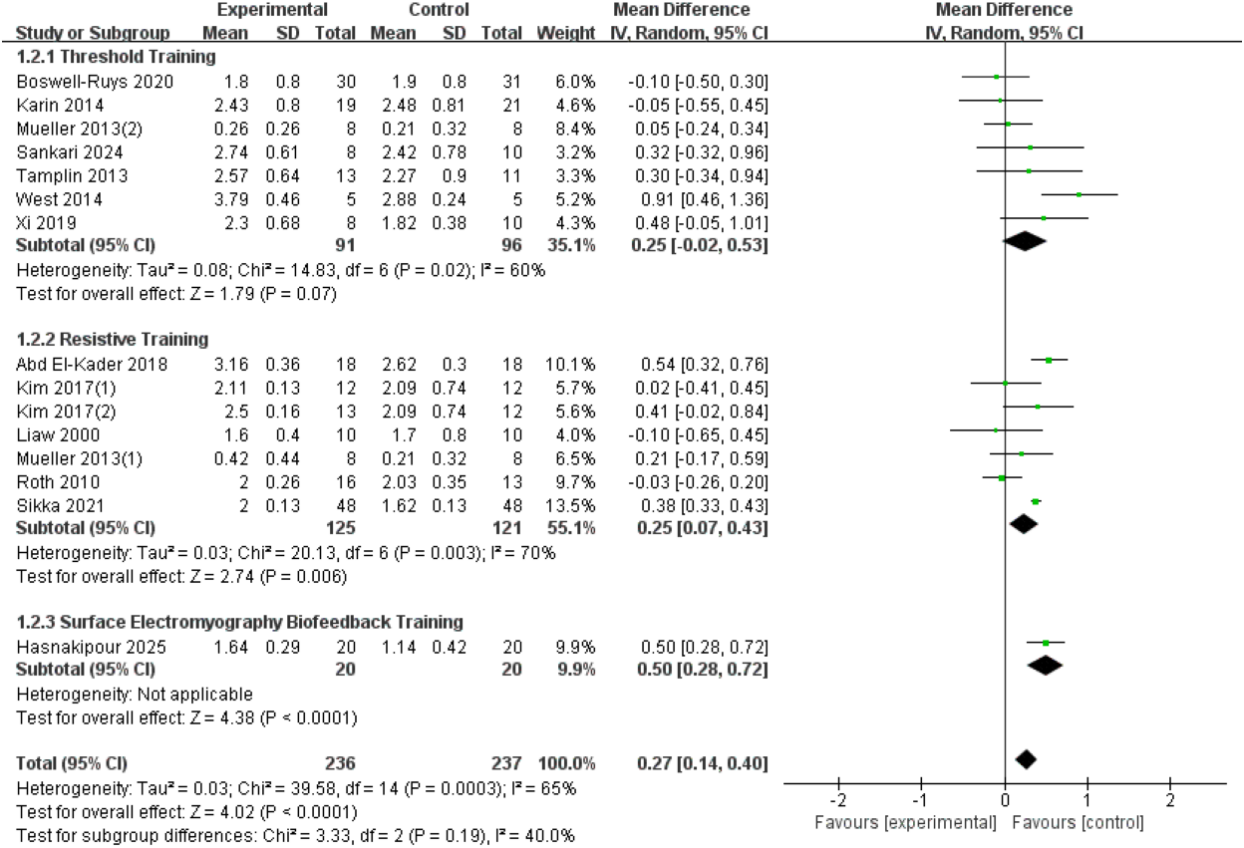
Forest plot of the meta-analysis on FEV_1_. Note. Boswell-Ruys et al., 2020; Postma et al., 2014; Mueller, Hopman & Perret, 2013; Sankari et al., 2024; Tamplin et al., 2013; West et al., 2014; Xi et al., 2019; Abd El-Kader, 2018; Kim et al., 2017; Liaw et al., 2000; Roth et al., 2010; Gitanjali et al., 2021.

A total of fifteen RCTs (Liaw et al., 2000; Roth et al., 2010; Mueller, Hopman & Perret, 2013; Tamplin et al., 2013; West et al., 2014; Postma et al., 2014; Kim et al., 2017; Abd El-Kader, 2018; Xi et al., 2019; Boswell-Ruys et al., 2020; Gitanjali et al., 2021; Sankari et al., 2024; Hasnakipour et al., 2025) with 473 patients were included in the analysis. Heterogeneity was present among the studies (*I*^2^ = 65%, *p* = 0.0003), leading to the use of a random-effects model. The results showed a significant improvement in the FEV_1_ index in SCI patients with RMT compared to the control group (*d* = 0.27, 95% CI [0.14∼0.4], *p* < 0.0001). We conducted subgroup analyses based on the type of intervention, categorizing the studies into three groups: threshold training, resistive training, and surface electromyography (sEMG) biofeedback training. The results showed:Threshold training did not lead to a statistically significant improvement in FEV_1_ (WMD = 0.25, 95% CI [−0.02–0.53], *p* = 0.07); resistive training significantly improved FEV1, with a statistically significant difference between the RMT group and the control group (WMD = 0.25, 95% CI [0.07–0.43], *p* = 0.007); sEMG biofeedback training showed the most significant improvement in FEV1 (WMD = 0.50, 95% CI [0.28–0.72], *p* < 0.0001). The subgroup difference test indicated no significant difference in FEV_1_ improvement between the three training modalities (Chi^2^ = 3.33, *df* = 2, *p* = 0.19) (Fig. 3).

#### FVC

**Figure 4:**
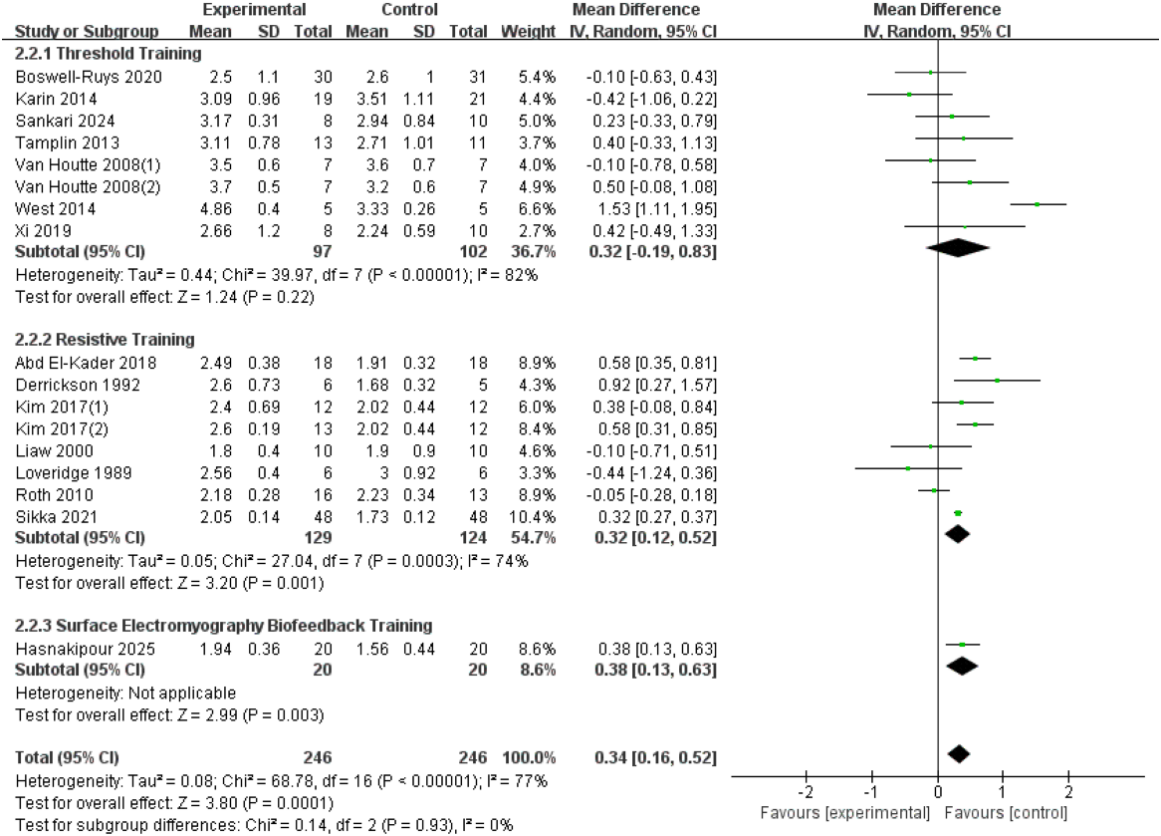
Forest plot of the meta-analysis on FVC. Note. Boswell-Ruys et al., 2020; Postma et al., 2014; Sankari et al., 2024; Tamplin et al., 2013; Van Houtte et al., 2008; West et al., 2014; Xi et al., 2019; Abd El-Kader, 2018; Derrickson et al., 1992; Kim et al., 2017; Liaw et al., 2000; Loveridge, Badour & Dubo, 1989; Roth et al., 2010; Gitanjali et al., 2021.

A total of seventeen RCTs (Loveridge, Badour & Dubo, 1989; Derrickson et al., 1992; Liaw et al., 2000; Van Houtte et al., 2008; Roth et al., 2010; Tamplin et al., 2013; West et al., 2014; Postma et al., 2014; Kim et al., 2017; Abd El-Kader, 2018; Xi et al., 2019; Boswell-Ruys et al., 2020; Gitanjali et al., 2021; Sankari et al., 2024; Hasnakipour et al., 2025) were included in the analysis, involving 492 patients with SCI. Heterogeneity was observed among the studies (*I*^2^ = 77%, *p* < 0.00001), and a random-effects model showed that RMT significantly improved FVC in SCI patients (*d* = 0.34, 95% CI [0.16 to −0.52], *p* = 0.0001). Subgroup analysis results indicate: Threshold training did not demonstrate a statistically significant improvement in the outcome measure (WMD = 0.32, 95% CI [−0.19–0.83], *p* = 0.22); resistive training showed a statistically significant improvement (WMD = 0.32, 95% CI [0.12–0.52], *p* = 0.001); sEMG biofeedback training also demonstrated a statistically significant effect (WMD = 0.38, 95% CI [0.13–0.63], *p* = 0.003). The subgroup difference test showed no significant distinction between the three training modalities in terms of FVC improvement (Chi^2^ = 0.14, *df* = 2, *p* = 00.93) (Fig. 4).

#### MIP

**Figure 5:**
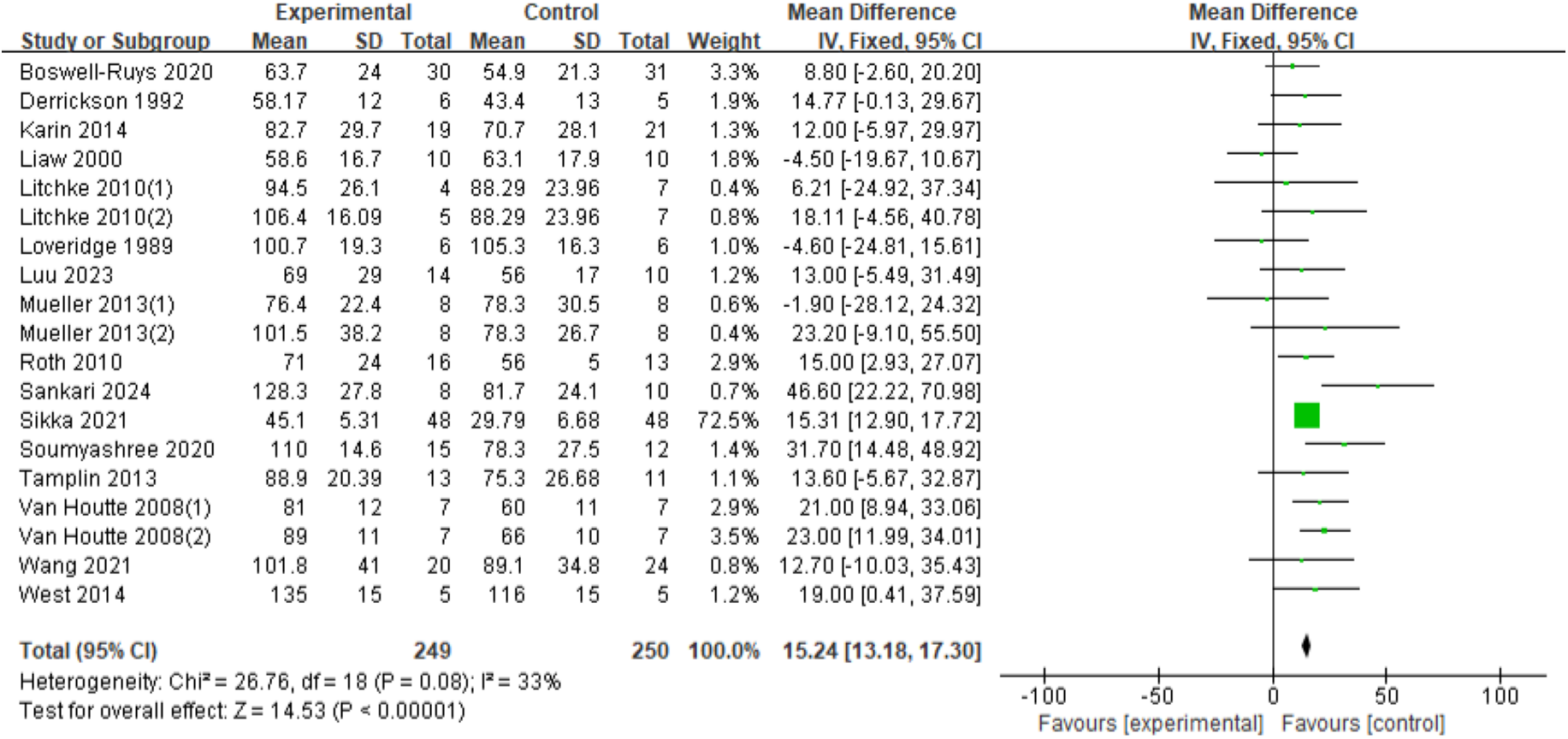
Forest plot of the meta-analysis on MIP. Note. Boswell-Ruys et al., 2020; Derrickson et al., 1992; Postma et al., 2014; Litchke et al., 2008; Loveridge, Badour & Dubo, 1989; Luu et al., 2023; Mueller, Hopman & Perret, 2013; Roth et al., 2010; Sankari et al., 2024; Gitanjali et al., 2021; Soumyashree & Kaur, 2020; Tamplin et al., 2013; Van Houtte et al., 2008; Wang et al., 2021; West et al., 2014.

In a total of nineteen RCTs (Loveridge, Badour & Dubo, 1989; Derrickson et al., 1992; Liaw et al., 2000; Van Houtte et al., 2008; Roth et al., 2010; Mueller, Hopman & Perret, 2013; Tamplin et al., 2013; West et al., 2014; Postma et al., 2014; Soumyashree & Kaur, 2020; Boswell-Ruys et al., 2020; Gitanjali et al., 2021; Wang et al., 2021; Luu et al., 2023; Sankari et al., 2024; Litchke et al., 2008) involving 499 patients, the fixed-effects model demonstrated a statistically significant improvement in MIP for SCI patients with RMT (*d* = 15.24, 95% CI [13.18∼17.30], *p* < 0.00001) (Fig. 5).

#### MEP

**Figure 6:**
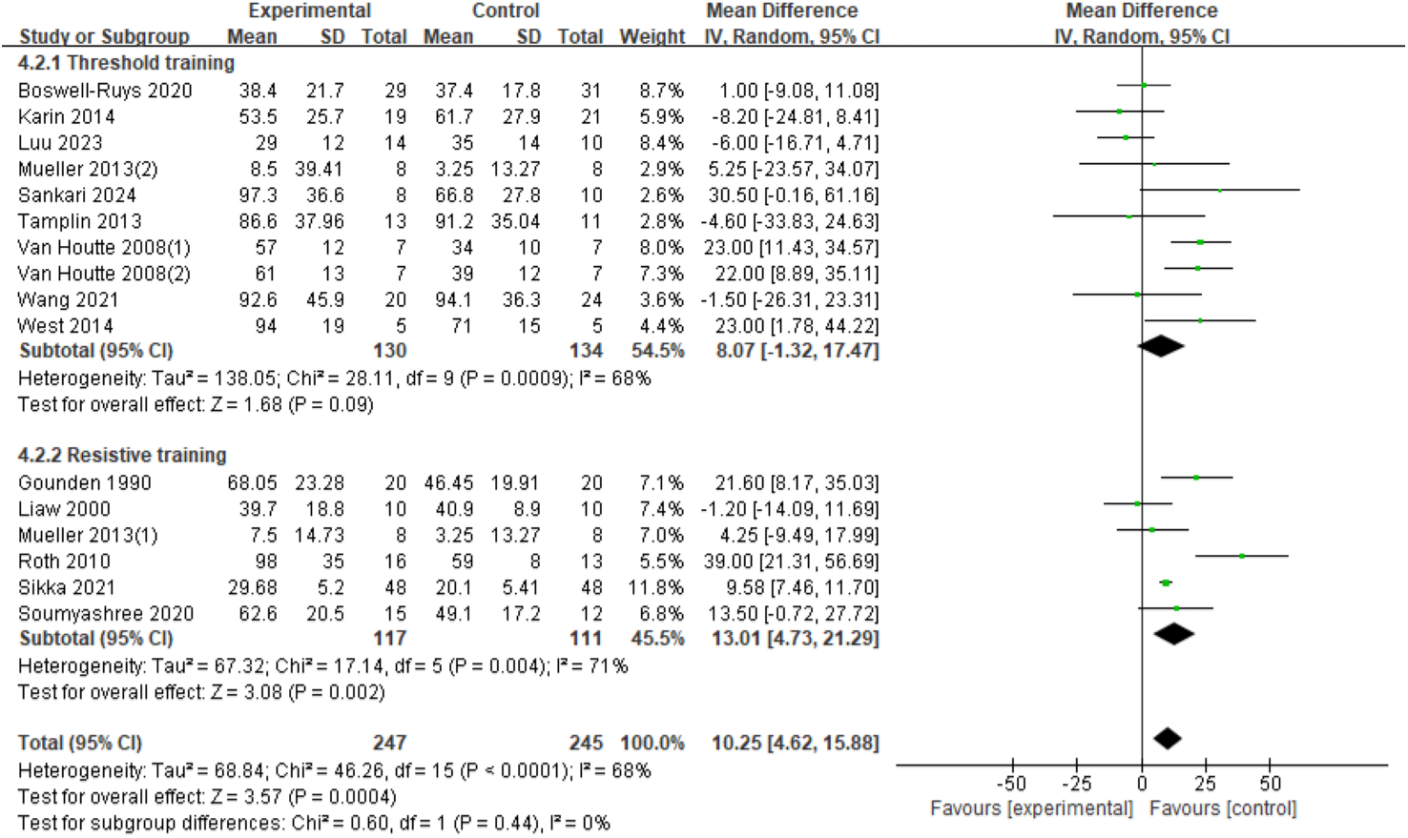
Forest plot of the meta-analysis on MEP. Note. Boswell-Ruys et al., 2020; Postma et al., 2014; Luu et al., 2023; Mueller, Hopman & Perret, 2013; Sankari et al., 2024; Tamplin et al., 2013; Van Houtte et al., 2008; Wang et al., 2021; West et al., 2014; Gounden, 1990; Liaw et al., 2000; Roth et al., 2010; Gitanjali et al., 2021; Soumyashree & Kaur, 2020.

Sixteen RCTs (Boswell-Ruys et al., 2020; Gitanjali et al., 2021; Gounden, 1990; Liaw et al., 2000; Luu et al., 2023; Mueller, Hopman & Perret, 2013; Postma et al., 2014; Roth et al., 2010; Sankari et al., 2024; Soumyashree & Kaur, 2020; Tamplin et al., 2013; Van Houtte et al., 2008; Wang et al., 2021; West et al., 2014) were analyzed, involving 492 patients. There was variation among the studies (*I*^2^ = 68%, *p* < 0.0001), leading to the use of a random-effects model. The results showed a significant improvement in the MEP index in SCI patients with RMT compared to control groups (*d* = 10.25, 95% CI [4.62∼15.88], *p* = 0.0004). Subgroup analyses were conducted, with the resistance training group showing a statistically significant improvement in MEP compared to the control group (*d* = 13.01, 95% CI [4.73∼21.29], *p* = 0.002). The threshold training group did not show a statistically significant improvement in MEP (*d* = 8.07, 95% CI [−1.32∼17.47], *p* = 0.09). The subgroup difference test indicated no significant difference between the two training modalities in terms of MEP improvement effect (Chi^2^ = 0.60, *df* = 1, *p* = 0.44) (Fig. 6).

#### PEF

**Figure 7:**
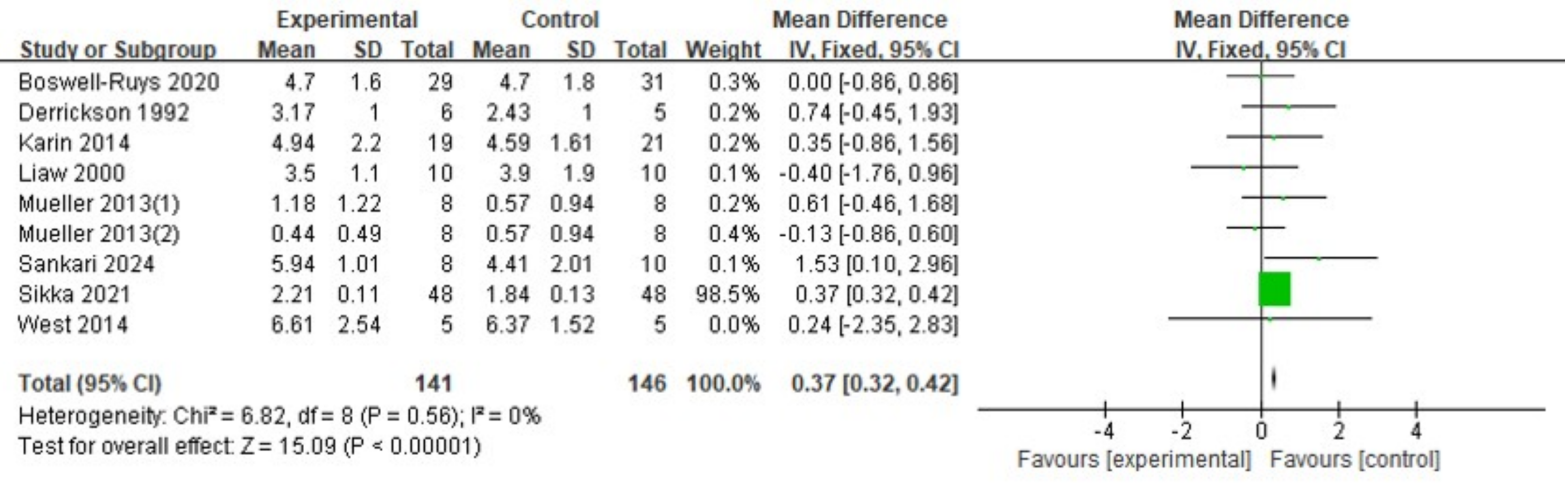
Forest plot of the meta-analysis on PEF. Note. Boswell-Ruys et al., 2020; Derrickson et al., 1992; Postma et al., 2014; Liaw et al., 2000; Mueller, Hopman & Perret, 2013; Sankari et al., 2024; Gitanjali et al., 2021; West et al., 2014.

The analysis included nine RCTs (Boswell-Ruys et al., 2020; Derrickson et al., 1992; Gitanjali et al., 2021; Liaw et al., 2000; Mueller, Hopman & Perret, 2013; Postma et al., 2014; Sankari et al., 2024; West et al., 2014) with 287 patients. The fixed-effects model indicated that RMT significantly increased PEF in SCI patients, with a statistically significant variance (*d* = 0.37, 95% CI [0.32 ∼0.42], *p* < 0.00001) (Fig. 7).

#### MVV

**Figure 8:**
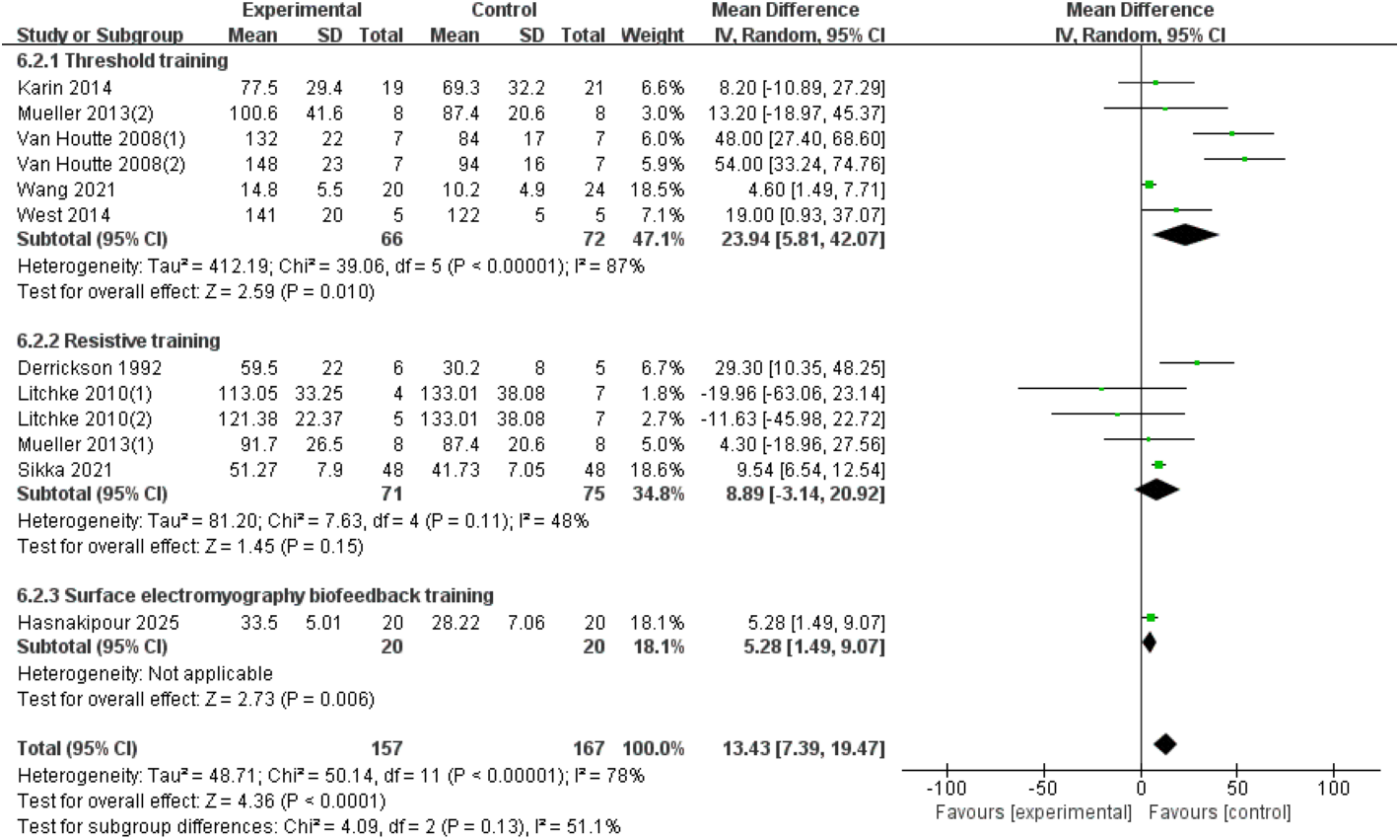
Forest plot of the meta-analysis on MVV. Note. Postma et al., 2014; Mueller, Hopman & Perret, 2013; Van Houtte et al., 2008; Wang et al., 2021; West et al., 2014; Derrickson et al., 1992; Litchke et al., 2008; Mueller, Hopman & Perret, 2013; Gitanjali et al., 2021.

Twelve RCTs (Derrickson et al., 1992; Van Houtte et al., 2008; Mueller, Hopman & Perret, 2013; West et al., 2014; Postma et al., 2014; Gitanjali et al., 2021; Wang et al., 2021; Hasnakipour et al., 2025; Litchke et al., 2008) were included, involving 324 patients, with heterogeneity present among the studies. A random-effects model was used due to high heterogeneity (*I*^2^ = 78%, *p* < 0.00001). The results showed a significant improvement in the MVV index for SCI patients with RMT compared to the control group (*d* = 15.97, 95% CI [8.37∼23.56], *p* < 0.0001). Subgroup analysis results indicate: The threshold training group showed a statistically significant improvement (WMD = 42.07, 95% CI [10.24–73.90], *p* = 0.010). The resistive training group did not show a statistically significant improvement (WMD = 8.89, 95% CI [−3.14–20.92], *p* = 0.15). The sEMG biofeedback training group showed a statistically significant effect (WMD = 5.28, 95% CI [1.49–9.07], *p* = 0.006). There was no significant difference between the three training modalities in terms of MVV improvement effect (Chi^2^ = 4.09, *df* = 2, *p* = 0.13) (Fig. 8).

#### TLC

**Figure 9:**
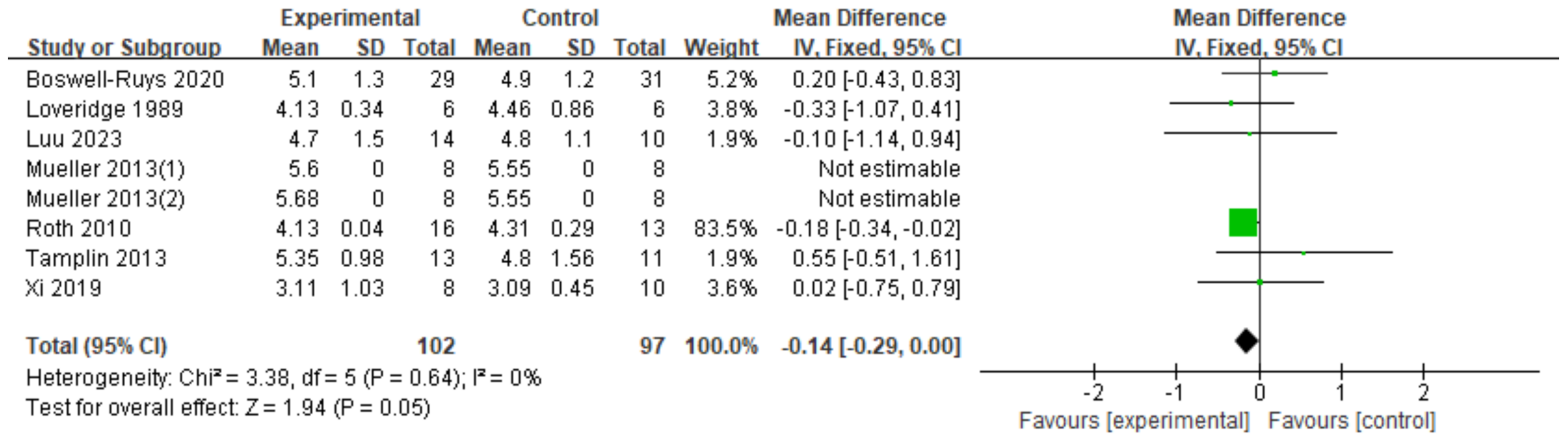
Forest plot of the meta-analysis on TLC. Note. Boswell-Ruys et al., 2020; Loveridge, Badour & Dubo, 1989; Luu et al., 2023; Mueller, Hopman & Perret, 2013; Roth et al., 2010; Tamplin et al., 2013; Xi et al., 2019.

Eight RCTs (Boswell-Ruys et al., 2020; Loveridge, Badour & Dubo, 1989; Luu et al., 2023; Mueller, Hopman & Perret, 2013; Roth et al., 2010; Tamplin et al., 2013; Xi et al., 2019), involving 199 patients, were analyzed. The results showed a small increase in TLC for SCI patients who received RMT, but the difference was not statistically significant. The gap between the two groups almost reached statistical significance (*d* = −0.14, 95% CI [−0.29∼0.00], *p* = 0.05) (Fig. 9).

#### IC

**Figure 10:**
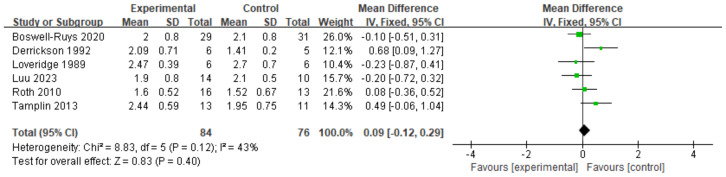
Forest plot of the meta-analysis on IC. Note. Boswell-Ruys et al., 2020; Derrickson et al., 1992; Loveridge, Badour & Dubo, 1989; Luu et al., 2023; Roth et al., 2010; Tamplin et al., 2013.

The analysis included six RCTs (Loveridge, Badour & Dubo, 1989; Derrickson et al., 1992; Roth et al., 2010; Tamplin et al., 2013; Boswell-Ruys et al., 2020; Luu et al., 2023) with a total of 161 patients. According to the fixed-effects model, RMT did not have a significant impact on IC in SCI patients. There was no statistically significant difference between the two groups (*d* = 0.09, 95% CI [−0.12∼0.29], *p* = 0.40) (Fig. 10).

#### VC

**Figure 11:**
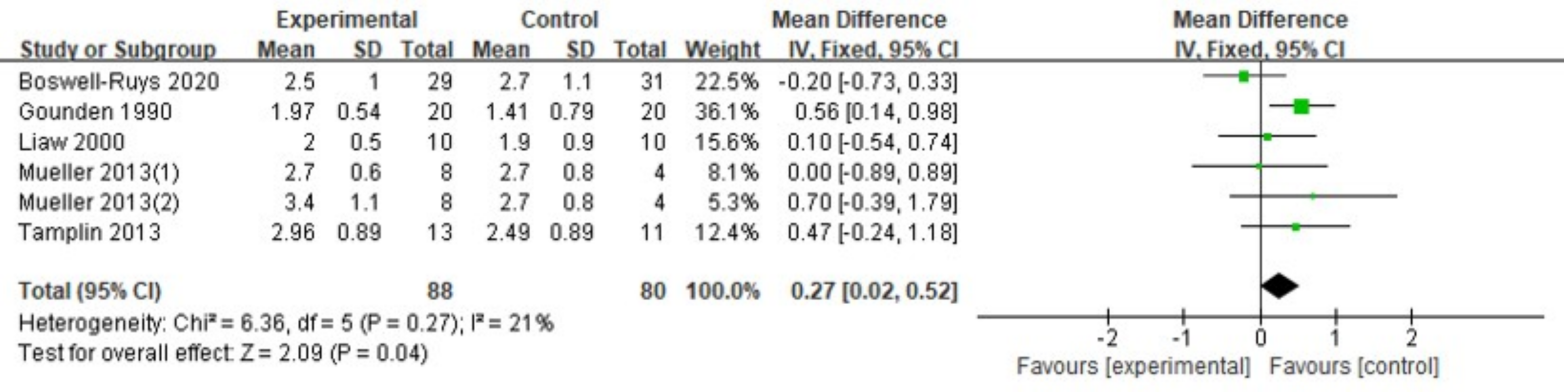
Forest plot of the meta-analysis on VC. Note. Boswell-Ruys et al., 2020; Gounden, 1990; Liaw et al., 2000; Mueller, Hopman & Perret, 2013; Tamplin et al., 2013.

In a review of six RCTs (Gounden, 1990; Liaw et al., 2000; Mueller, Hopman & Perret, 2013; Tamplin et al., 2013; Boswell-Ruys et al., 2020) involving 168 patients, it was found that RMT resulted in a slight increase in VC for individuals with SCI. The RMT group outperformed the control group in enhancing VC (*d* = 0.27, 95% CI [0.02∼0.52], *p* = 0.04) (Fig. 11).

### Publication bias

**Figure 12:**
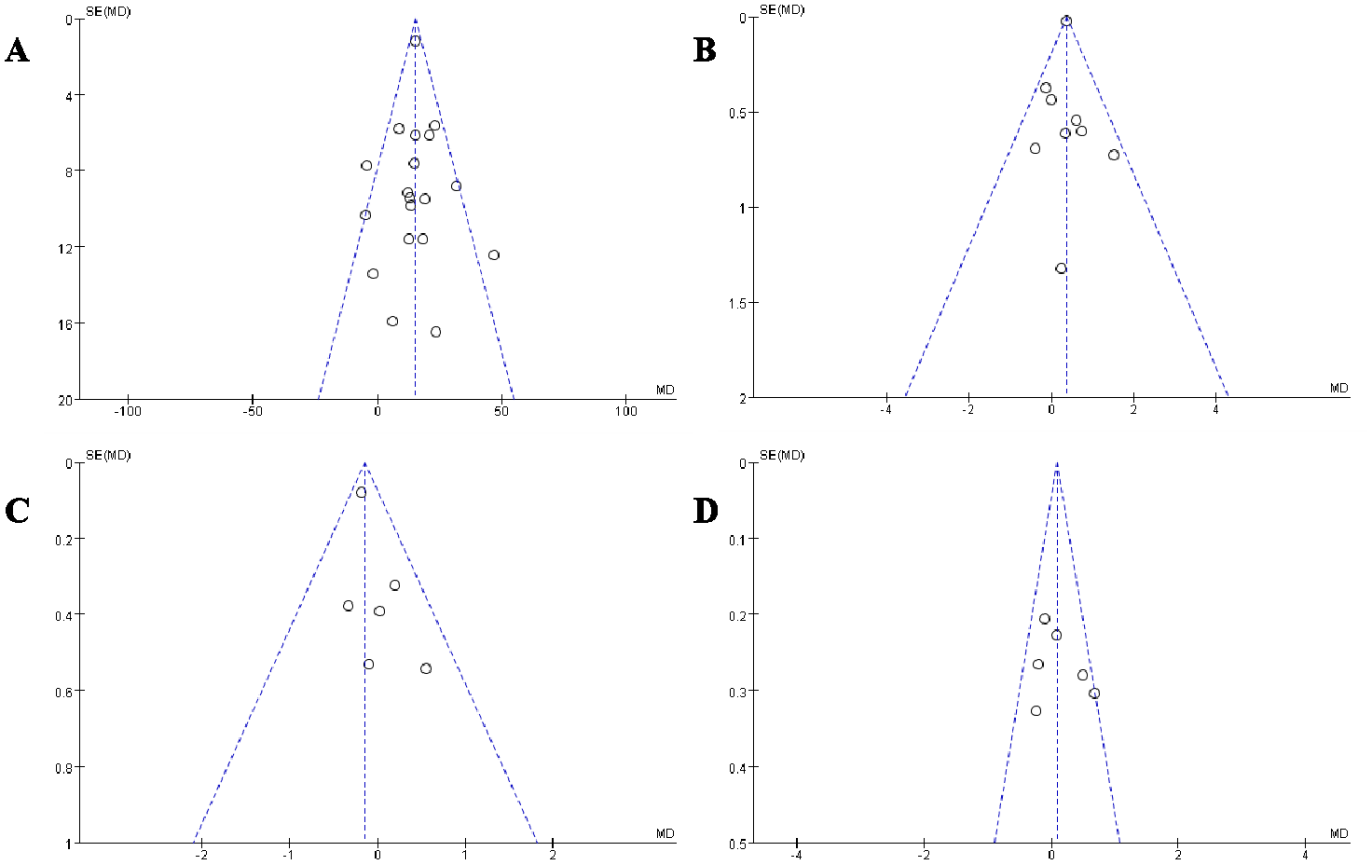
Publication bias funnel plots.

In this meta-analysis, we selected four key indicators for detailed funnel plot analysis: MIP, PEF, TLC, and IC (Fig. 12). The scatter plots and funnel plots of the outcome metrics in the included studies showed a balanced distribution from left to right, indicating no publication bias. The stability and reliability of the results of the meta-analysis in this study were confirmed, as both the sensitivity analysis of the included studies and the literature-by-exclusion had no significant impact on the outcome indicators.

## Discussion

The global prevalence of SCI increased by 81.5% from 1990 to 2019, with the number of individuals living with SCI surpassing 20 million in 2019. There were an estimated 900,000 new cases of SCI recorded in that year (GBD Spinal Cord Injuries Collaborators, 2023). SCI has become a significant health concern worldwide (Sterner & Sterner, 2022), leading to severe physical and psychological consequences (Rodrigues et al., 2025). Respiratory dysfunction due to SCI is a major cause of morbidity, mortality, and economic burden, resulting in a higher mortality rate in SCI patients compared to the general population (Boswell-Ruys et al., 2015). Research has shown the potential benefits of RMT in individuals with SCI (Berlowitz, Wadsworth & Ross, 2016; Gee & West, 2018; Kang, Park & Eun, 2022; McDonald & Stiller, 2019; Ushiku et al., 2019), highlighting the importance of promoting its use in clinical settings to improve the well-being and quality of life of these patients.

This study systematically evaluated and conducted a meta-analysis of RCTs on RMT for SCI patients, identifying 25 relevant RCTs. By extracting data and combining effect sizes, the literature was analyzed to assess the impact of RMT on lung function recovery in SCI patients. The analysis revealed that RMT led to significant improvements in FEV1, FVC, MIP, MEP, PEF, MVV, TLC, and VC functions compared to the control group among SCI patients, consistent with findings from various studies (Gitanjali et al., 2021; Kang, Park & Eun, 2022; Sankari et al., 2024). Normocapnic hyperventilation training has been shown to have a significant positive impact on lung function in patients with SCI (Xi et al., 2019). Patients who underwent RMT demonstrated improved lung function (FVC, FEV_1_, MVV) compared to those in the control group. RMT resulted in enhancements in respiratory function (FVC, FEV_1_, PEFR, SVC, MVV) and respiratory strength (MIP and MEP) in patients with complete cervical cord injuries, even up to 1 month post-injury (Sankari et al., 2024). Game-based RMT was effective in individuals with chronic cervical SCI, leading to improvements in lung function and muscle strength (MIP and MEP) (Kang, Park & Eun, 2022). RMT also significantly increased MIP in individuals with acute cervical SCI (Wang et al., 2021). Additionally, RMT improved MIP and MEP for tetraplegic patients, along with enhancements in FVC, FEV1, PEF, and MVV (Gitanjali et al., 2021). RMT is a successful method for addressing respiratory dysfunction in various populations, enhancing respiratory muscle strength, endurance, and coordination (Rice et al., 2020; Zhang et al., 2020; Ramli et al., 2023). Given that individuals with SCI often experience pulmonary issues due to compromised respiratory muscle function, RMT is a valuable intervention for restoring lung function by improving breathing patterns and respiratory efficiency.

Our study found no significant difference in inspiratory capacity between the RMT group and the control group, contrary to Tamplin’s findings (Tamplin & Berlowitz, 2014). This could be due to the specificity of RMT focusing on the strength and endurance of respiratory muscles, while improvement in inspiratory capacity may rely more on lung elasticity and thoracic compliance. Additionally, inadequate intensity and duration of training in some studies may have hindered the improvement of inspiratory capacity. Furthermore, individual differences in respiratory muscle function and rehabilitation potential among patients may result in varied responses to RMT.

Our subgroup analyses of different RMT modalities—threshold, resistive, and sEMG biofeedback—yielded nuanced results. While the overall tests for subgroup differences were not statistically significant, likely due to limited power, certain trends emerged. For FEV_1_ and FVC, resistive training and sEMG biofeedback showed significant effects, whereas threshold training did not. Conversely, for MVV, threshold training and sEMG biofeedback were effective, but resistive training was not. This suggests that different training mechanisms may target different aspects of respiratory function. For instance, sEMG biofeedback may enhance neuromuscular control and coordination, while resistive training may be more effective for building pure strength. A recent network meta-analysis also highlighted that different respiratory training methods have varying efficacy for specific outcomes like MIP, FEV_1_, and FVC (Chen et al., 2025). The principle that targeted training improves function is well-established across various populations, including individuals post-stroke (Fabero-Garrido et al., 2022), patients with chronic obstructive pulmonary diseas (Huang et al., 2024), and even healthy athletes (HajGhanbari et al., 2013), lending further support to our findings.

The clinical significance of these findings is substantial. Improved respiratory muscle function can lead to a reduced incidence of respiratory complications like pneumonia and atelectasis, which are primary causes of re-hospitalization and mortality in SCI patients (Sezer, 2015). Enhanced cough efficacy, stemming from stronger expiratory muscles, is critical for airway hygiene (McBain et al., 2013). Furthermore, improved respiratory endurance can contribute to increased overall physical activity levels and participation in daily life, thereby enhancing quality of life (Boswell-Ruys et al., 2020). The study by Burtin et al. (2009), although in critically ill patients, highlights how enhanced functional capacity can improve outcomes. The implications extend to reducing healthcare costs associated with managing respiratory complications (Berlly & and Shem, 2007).

### Study limitations

Even though the results of meta-analysis in this study support the validity of RMT, certain limitations were identified during the study. (a) Some studies included in the analysis had issues with bias assessment, such as inadequate randomization methods and blinding, which may have impacted the accuracy of the results; (b) variations in the methods, intensity, and duration of RMT in different studies could have influenced the results of the meta-analysis; (c) small sample sizes in some studies may have limited the generalizability of the results; (d) most included studies had low to moderate quality reporting; (e) the predominance of older trials among the included studies may limit the contemporary relevance of the evidence base. Based on the limitations of existing research and the timeliness of the evidence, future studies should focus on the following directions: first, it is essential to thoroughly explore optimal implementation strategies for respiratory muscle training, systematically evaluating the optimal combination of parameters such as training type, intensity, frequency, and duration. Second, active investigation into combined intervention models integrating RMT with other rehabilitation tools should be pursued to synergistically enhance rehabilitation outcomes. Additionally, long-term follow-up studies are necessary to clarify the sustained effects of RMT and patient compliance, ensuring its sustainable application in clinical practice. Finally, there is an urgent need for more recent high-quality studies to update the existing evidence base and validate the timeliness of current conclusions.

## Conclusion

In summary, this meta-analysis provides strong evidence that RMT is a safe and effective intervention for improving respiratory muscle strength and key measures of pulmonary function in individuals with SCI. The findings robustly support the inclusion of RMT in standard rehabilitation programs to address respiratory muscle weakness and its functional consequences. Despite the clear benefits, significant heterogeneity across studies highlights the need for further high-quality research to refine training protocols, compare different modalities, and establish the optimal application of RMT to maximize clinical outcomes and enhance the quality of life for this vulnerable population.

## Supplemental Information

10.7717/peerj.20373/supp-1 Supplemental Information 1 PRISMA checklist

10.7717/peerj.20373/supp-2 Supplemental Information 2 Basic characteristics of the included studies

10.7717/peerj.20373/supp-3 Supplemental Information 3 Intervention Characteristics for Respiratory Muscle Training in Spinal Cord Injury Patients

10.7717/peerj.20373/supp-4 Supplemental Information 4 Physiotherapy Evidence Database score of the included studies

10.7717/peerj.20373/supp-5 Supplemental Information 5 Search Strategy and Data

## Additional Information and Declarations

### Data Availability

The following information was supplied regarding data availability: The raw data is available in the Supplemental Files.

## References

[ref-1] Abd El-Kader SM (2018). Impact of respiratory muscle training on blood gases and pulmonary function among patients with cervical spinal cord injury. Electronic Journal of General Medicine.

[ref-2] Albanese E, Bütikofer L, Armijo-Olivo S, Ha C, Egger M (2020). Construct validity of the Physiotherapy Evidence Database (PEDro) quality scale for randomized trials: item response theory and factor analyses. Research Synthesis Methods.

[ref-3] Arora S, Flower O, Murray NPS, Lee BB (2012). Respiratory care of patients with cervical spinal cord injury: a review. Critical Care and Resuscitation: Journal of the Australasian Academy of Critical Care Medicine.

[ref-4] Berlly MDM, Shem MDK (2007). Respiratory management during the first five days after spinal cord injury. The Journal of Spinal Cord Medicine.

[ref-5] Berlowitz DJ, Tamplin J (2013). Respiratory muscle training for cervical spinal cord injury. The Cochrane Database of Systematic Reviews.

[ref-6] Berlowitz DJ, Wadsworth B, Ross J (2016). Respiratory problems and management in people with spinal cord injury. Breathe.

[ref-7] Boswell-Ruys CL, Lewis CRH, Gandevia SC, Butler JE (2015). Respiratory muscle training may improve respiratory function and obstructive sleep apnoea in people with cervical spinal cord injury. Spinal Cord Series and Cases.

[ref-8] Boswell-Ruys CL, Lewis CRH, Wijeysuriya NS, McBain RA, Lee BB, McKenzie DK, Gandevia SC, Butler JE (2020). Impact of respiratory muscle training on respiratory muscle strength, respiratory function and quality of life in individuals with tetraplegia: a randomised clinical trial. Thorax.

[ref-9] Bott J, Blumenthal S, Buxton M, Ellum S, Falconer C, Garrod R, Harvey A, Hughes T, Lincoln M, Mikelsons C, Potter C, Pryor J, Rimington L, Sinfield F, Thompson C, Vaughn P, White J (2009). Guidelines for the physiotherapy management of the adult, medical, spontaneously breathing patient. Thorax.

[ref-10] Brown R, Di Marco AF, Hoit JD, Garshick E (2006). Respiratory dysfunction and management in spinal cord injury. Respiratory Care.

[ref-11] Burtin C, Clerckx B, Robbeets C, Ferdinande P, Langer D, Troosters T, Hermans G, Decramer M, Gosselink R (2009). Early exercise in critically ill patients enhances short-term functional recovery*. Critical Care Medicine.

[ref-12] Chen J, Chen T, Wang X, Tang X, Yang S, Lyu K, Li F, Xian J, Tan B, Chen Y (2025). Effects of different respiratory training methods on respiratory function in patients with spinal cord injury: a network meta-analysis of randomized controlled trials. Critical Care.

[ref-13] Choi EH, Gattas S, Brown NJ, Hong JD, Limbo JN, Chan AY, Oh MY (2021). Epidural electrical stimulation for spinal cord injury. Neural Regeneration Research.

[ref-14] Courtine G, Sofroniew MV (2019). Spinal cord repair: advances in biology and technology. Nature Medicine.

[ref-15] Derrickson J, Ciesla N, Simpson N, Imle PC (1992). A comparison of two breathing exercise programs for patients with quadriplegia. Physical Therapy.

[ref-16] Fabero-Garrido R, Corral Tdel, Angulo-Díaz-Parreño S, Plaza-Manzano G, Martín-Casas P, Cleland JA, Fernández-de-las Peñas C, López-de Uralde-Villanueva I (2022). Respiratory muscle training improves exercise tolerance and respiratory muscle function/structure post-stroke at short term: a systematic review and meta-analysis. Annals of Physical and Rehabilitation Medicine.

[ref-17] Fabero-Garrido R, del Corral T, Plaza-Manzano G, Sanz-Ayan P, Izquierdo-García J, López-de Uralde-Villanueva I (2024). Effects of respiratory muscle training on exercise capacity, quality of life, and respiratory and pulmonary function in people with ischemic heart disease: systematic review and meta-analysis. Physical Therapy.

[ref-18] GBD Spinal Cord Injuries Collaborators (2023). Global, regional, and national burden of spinal cord injury, 1990-2019: a systematic analysis for the Global Burden of Disease Study 2019. The Lancet. Neurology.

[ref-19] Gee CM, West CR (2018). Effects of respiratory training on heart rate variability and baroreflex sensitivity in individuals with chronic spinal cord injury. Archives of Physical Medicine and Rehabilitation.

[ref-20] Gitanjali S, Joginder Y, Roop S, Gupta KB (2021). Effect of 4 weeks resistive inspiratory muscle training on respiratory functions in patients with tetraplegia during in-patient rehabilitation. International Journal of Research in Pharmaceutical Sciences.

[ref-21] Gounden P (1990). Progressive resistive loading on accessory expiratory muscles in tetraplegia. South African Journal of Physiotherapy.

[ref-22] HajGhanbari B, Yamabayashi C, Buna TR, Coelho JD, Freedman KD, Morton TA, Palmer SA, Toy MA, Walsh C, Sheel AW, Reid WD (2013). Effects of respiratory muscle training on performance in athletes: a systematic review with meta-analyses. The Journal of Strength & Conditioning Research.

[ref-23] Hasnakipour S, Mosallanezhad Z, Rezaeian T, Azadi F, Noroozi M (2025). Effect of diaphragmatic breathing exercise with biofeedback on respiratory function in incomplete cervical spinal cord injury: a randomized-controlled study. American Journal of Physical Medicine & Rehabilitation.

[ref-24] Higgins JPT, Altman DG, Gøtzsche PC, Jüni P, Moher D, Oxman AD, Savovic J, Schulz KF, Weeks L, Sterne JAC, Cochrane Bias Methods Group, Cochrane Statistical Methods Group (2011). The Cochrane Collaboration’s tool for assessing risk of bias in randomised trials. BMJ.

[ref-25] Huang Z, Li Z, Yan M, Zheng J, Huang W, Hong L, Lu Q, Liu L, Huang X, Fan H, Su W, Huang X, Wu X, Guo Z, Qiu C, Zhao Z, Hong Y (2024). Effect of respiratory muscle training in patients with stable chronic obstructive pulmonary disease: a systematic review and meta-analysis. Heliyon.

[ref-26] Jackson AB, Groomes TE (1994). Incidence of respiratory complications following spinal cord injury. Archives of Physical Medicine and Rehabilitation.

[ref-27] Kang D, Park J, Eun S-D (2022). A preliminary study on the feasibility of community game-based respiratory muscle training for individuals with high cervical spinal cord injury levels: a novel approach. BMC Sports Science, Medicine & Rehabilitation.

[ref-28] Kim C-Y, Lee J-S, Kim H-D, Lee D-J (2017). Short-term effects of respiratory muscle training combined with the abdominal drawing-in maneuver on the decreased pulmonary function of individuals with chronic spinal cord injury: a pilot randomized controlled trial. The Journal of Spinal Cord Medicine.

[ref-29] Kumru H, García-Alén L, Ros-Alsina A, Albu S, Valles M, Vidal J (2023). Transcutaneous spinal cord stimulation improves respiratory muscle strength and function in subjects with cervical spinal cord injury: original research. Biomedicines.

[ref-30] Liaw MY, Lin MC, Cheng PT, Wong MK, Tang FT (2000). Resistive inspiratory muscle training: its effectiveness in patients with acute complete cervical cord injury. Archives of Physical Medicine and Rehabilitation.

[ref-31] Litchke L, Lloyd L, Schmidt E, Russian C, Reardon RF (2008). Comparison of two concurrent respiratory resistance devices on pulmonary function and time trial performance of wheelchair athletes. Respiratory Care.

[ref-32] Lorach H, Galvez A, Spagnolo V, Martel F, Karakas S, Intering N, Vat M, Faivre O, Harte C, Komi S, Ravier J, Collin T, Coquoz L, Sakr I, Baaklini E, Hernandez-Charpak SD, Dumont G, Buschman R, Buse N, Denison T, Van Nes I, Asboth L, Watrin A, Struber L, Sauter-Starace F, Langar L, Auboiroux V, Carda S, Chabardes S, Aksenova T, Demesmaeker R, Charvet G, Bloch J, Courtine G (2023). Walking naturally after spinal cord injury using a brain-spine interface. Nature.

[ref-33] Loveridge B, Badour M, Dubo H (1989). Ventilatory muscle endurance training in quadriplegia: effects on breathing pattern. Paraplegia.

[ref-34] Luu BL, Lewis RHC, McBain RA, Gandevia SC, Boswell-Ruys CL, Butler JE (2023). Effect of respiratory muscle training on load sensations in people with chronic tetraplegia: a secondary analysis of a randomised controlled trial. Spinal Cord.

[ref-35] Maher CG, Sherrington C, Herbert RD, Moseley AM, Elkins M (2003). Reliability of the PEDro scale for rating quality of randomized controlled trials. Physical Therapy.

[ref-36] McBain RA, Boswell-Ruys CL, Lee BB, Gandevia SC, Butler JE (2013). Abdominal muscle training can enhance cough after spinal cord injury. Neurorehabilitation and Neural Repair.

[ref-37] McDonald T, Stiller K (2019). Inspiratory muscle training is feasible and safe for patients with acute spinal cord injury. The Journal of Spinal Cord Medicine.

[ref-38] Mohammadi E, Villeneuve LM, Smith ZA (2023). Spinal cord injury: the global incidence, prevalence, and disability from the global burden of disease study 2019. Spine.

[ref-39] Moher D, Liberati A, Tetzlaff J, Altman DG, P RG (2009). Preferred reporting items for systematic reviews and meta-analyses: the PRISMA statement. PLOS Medicine.

[ref-40] Moreno MA, Zamunér AR, Paris JV, Teodori RM, Barros RML (2012). Effects of wheelchair sports on respiratory muscle strength and thoracic mobility of individuals with spinal cord injury. American Journal of Physical Medicine & Rehabilitation.

[ref-41] Mueller G, Hopman MTE, Perret C (2013). Comparison of respiratory muscle training methods in individuals with motor and sensory complete tetraplegia: a randomized controlled trial. Journal of Rehabilitation Medicine.

[ref-42] Postma K, Haisma JA, Hopman MTE, Bergen MP, Stam HJ, Bussmann JB (2014). Resistive inspiratory muscle training in people with spinal cord injury during inpatient rehabilitation: a randomized controlled trial. Physical Therapy.

[ref-43] Ramli MI, Hamzaid NA, Engkasan JP, Usman J (2023a). Respiratory muscle training: a bibliometric analysis of 60 years’ multidisciplinary journey. Biomedical Engineering Online.

[ref-44] Rice H, Harrold M, Fowler R, Watson C, Waterer G, Hill K (2020). Exercise training for adults hospitalized with an acute respiratory condition: a systematic scoping review. Clinical Rehabilitation.

[ref-45] Rodrigues FO, Padovani MMP, Lopes BP, De Moura JA, Gama ACC (2025). Respiratory muscle training in people with cervical spinal cord injury - a systematic review. The Journal of Spinal Cord Medicine.

[ref-46] Roth EJ, Stenson KW, Powley S, Oken J, Primack S, Nussbaum SB, Berkowitz M (2010). Expiratory muscle training in spinal cord injury: a randomized controlled trial. Archives of Physical Medicine and Rehabilitation.

[ref-47] Rutchik A, Weissman AR, Almenoff PL, Spungen AM, Bauman WA, Grimm DR (1998). Resistive inspiratory muscle training in subjects with chronic cervical spinal cord injury. Archives of Physical Medicine and Rehabilitation.

[ref-48] Sankari A, Najjar AA, Maresh SA, Prowting JL, Fung CH, Knack A, Yarandi H, Badr MS (2024). Feasibility of oropharyngeal and respiratory muscle training in individuals with OSA and spinal cord injury or disease: a pilot study. Physiological Reports.

[ref-49] Sezer N (2015). Chronic complications of spinal cord injury. World Journal of Orthopedics.

[ref-50] Shin JC, Han EY, Cho KH, Im SH (2019). Improvement in pulmonary function with short-term rehabilitation treatment in spinal cord injury patients. Scientific Reports.

[ref-51] Skinnider MA, Gautier M, Teo AYY, Kathe C, Hutson TH, Laskaratos A, De Coucy A, Regazzi N, Aureli V, James ND, Schneider B, Sofroniew MV, Barraud Q, Bloch J, Anderson MA, Squair JW, Courtine G (2024). Single-cell and spatial atlases of spinal cord injury in the Tabulae Paralytica. Nature.

[ref-52] Soumyashree S, Kaur J (2020). Effect of inspiratory muscle training (IMT) on aerobic capacity, respiratory muscle strength and rate of perceived exertion in paraplegics. The Journal of Spinal Cord Medicine.

[ref-53] Sterner RC, Sterner RM (2022). Immune response following traumatic spinal cord injury: Pathophysiology and therapies. Frontiers in Immunology.

[ref-54] Tamplin J, Baker FA, Grocke D, Brazzale DJ, Pretto JJ, Ruehland WR, Buttifant M, Brown DJ, Berlowitz DJ (2013). Effect of singing on respiratory function, voice, and mood after quadriplegia: a randomized controlled trial. Archives of Physical Medicine and Rehabilitation.

[ref-55] Tamplin J, Berlowitz DJ (2014). A systematic review and meta-analysis of the effects of respiratory muscle training on pulmonary function in tetraplegia. Spinal Cord.

[ref-56] Templeman L, Roberts F (2020). Effectiveness of expiratory muscle strength training on expiratory strength, pulmonary function and cough in the adult population: a systematic review. Physiotherapy.

[ref-57] Turcios NL (2020). Cystic fibrosis lung disease: an overview. Respiratory Care.

[ref-58] Ushiku C, Suda K, Matsumoto S, Komatsu M, Takahata M, Iwasaki N, Minami A (2019). Time course of respiratory dysfunction and motor paralysis for 12 weeks in cervical spinal cord injury without bone injury. Spine Surgery and Related Research.

[ref-59] Van Houtte S, Vanlandewijck Y, Kiekens C, Spengler CM, Gosselink R (2008). Patients with acute spinal cord injury benefit from normocapnic hyperpnoea training. Journal of Rehabilitation Medicine.

[ref-60] Wang H-C, Lin Y-T, Huang C-C, Lin M-C, Liaw M-Y, Lu C-H (2021). Effects of respiratory muscle training on baroreflex sensitivity, respiratory function, and serum oxidative stress in acute cervical spinal cord injury. Journal of Personalized Medicine.

[ref-61] West CR, Taylor BJ, Campbell IG, Romer LM (2014). Effects of inspiratory muscle training on exercise responses in Paralympic athletes with cervical spinal cord injury. Scandinavian Journal of Medicine & Science in Sports.

[ref-62] Xi J, Jiang H, Zhang N, Wang J, Zhang B, Cao H, Yang B, Frerichs I, Möller K, Zhao Z (2019). Respiratory muscle endurance training with normocapnic hyperpnoea for patients with chronic spinal cord injury: a pilot short-term randomized controlled trial. Journal of Rehabilitation Medicine.

[ref-63] Zhang X, Zheng Y, Dang Y, Wang L, Cheng Y, Zhang X, Mao M, Lu X (2020). Can inspiratory muscle training benefit patients after stroke? A systematic review and meta-analysis of randomized controlled trials. Clinical Rehabilitation.

[ref-64] Zheng L, Sun H, Chen Q, Xie X, Jin H, Ding Y (2025). Influential factors of adherence to inhalation drug therapy in patients with stable chronic obstructive pulmonary disease. Journal of Evaluation in Clinical Practice.

