# Supplementary material for: Effectiveness of respiratory muscle training on pulmonary function recovery in patients with spinal cord injury: a systematic review and meta-analysis
Source: PeerJ. 2025 Nov 28;13:e20373. doi: 10.7717/peerj.20373 (PMC12667691; doi:10.7717/peerj.20373)
Supplement: Supplemental Information 2 [file peerj-13-20373-s002.docx]

| Author, year | Study Area | Sample Size | | Age | | intervention | | outcomes |
| --- | --- | --- | --- | --- | --- | --- | --- | --- |
|  |  | T | C | T | C | T | C |  |
| Hasnakipour (2025) | Iran | 20 | 20 | 33.10 ± 4.80 | 32.25 ± 5.43 | RMT (Surface Electromyography Biofeedback Training) | Conventional intervention | FEV_1_, FVC, MVV |
| Sankari (2024) | United States | 8 | 10 | 66.3±10.0 | 64.8±7.7 | RMT+Oropharyngeal training (Threshold training) | Sham therapy | FEV_1_,FVC,MIP,MEP,PEF |
| Luu (2023) | Australia | 14 | 10 | 54±9.0 | 54±11.0 | RMT (Threshold training) | Sham therapy | MIP,MEP,TLC,IC |
| Wang (2021) | China | 20 | 24 | 46.1±14.0 | 44.8 ± 15.5 | RMT (Dofin Respiratory Trainer) | Sham therapy | MIP,MEP,MVV |
| Sikka (2021) | India | 48 | 45 | 39.54±13.08 | 42.42±10.97 | RMT (Resistance training) | Conventional intervention | FEV_1_,FVC,MIP,MEP,PEF,MVV |
| Boswell-Ruys (2020) | Australia | 30 | 31 | 51.5±14.3 | 55.7±14.9 | RMT (Threshold training) | Sham therapy | FEV_1_,FVC,MIP,MEP,PEF,TLC,IC,VC |
| Xi (2019) | China | 8 | 10 | 54.3±6.6 | 52.9±8.0 | RMT (Carbon dioxide ventilation ) | Conventional intervention | FEV_1_,FVC,TLC |
| Abd El-Kader (2018) | Egypt | 18 | 18 | 31.24±7.37 | 29.95±6.14 | RMT (Resistance training) | Conventional intervention | FEV_1_,FVC |
| Kim (1)(2017) | Korea | 12 | 13 | 39.98±11.47 | 40.12±8.73 | RMT (Resistance training) | Conventional intervention | FEV_1_,FVC |
| Kim (2)(2017) | Korea | 12 | 12 | 41.51±10.04 | 40.12±8.73 | RMT (Stabilizers, abdominal traction training) | Conventional intervention | FEV_1_,FVC |
| Postma (2014) | Netherlands | 19 | 21 | 47.1±14.1 | 46.6±14.9 | RMT (Threshold training) | Conventional intervention | FEV_1_,FVC,MIP,MEP,PEF,MVV |
| West (2014) | United Kingdom | 5 | 5 | 30.5±2.2 | 27.9±2.8 | RMT (Threshold training) | Placebo | FEV_1_,FVC,MIP,MEP,PEF,MVV |
| Roth (2010) | United States | 16 | 13 | 31.1±12.4 | 28.9±9.6 | RMT (Resistance training) | Sham therapy | FEV_1_,FVC,MIP,MEP,TLC,IC |
| Liaw (2000) | China | 10 | 10 | 30.9±11.6 | 36.5± 11.5 | RMT (DHD respiratory trainer) | Conventional intervention | FEV_1_,FVC,MIP,MEP, PEF,VC |
| Loveridge(1989) | Canada | 6 | 6 | 31±4.1 | 35±12 | RMT (Resistance training) | Conventional intervention | FVC,MIP,TLC,IC |
| Derrickson(1992) | United States | 6 | 5 | 28.5±5.6 | 27±10.7 | RMT (Resistance training) | Abdomen Weight Training | FVC,MIP ,PEF,MVV, IC |
| Mueller(1)(2013) | Switzerland | 8 | 8 | 35.2±12.7 | 41.6±17 | RMT (Respifit S instrument) | Placebo | FEV_1_,MIP,MEP,PEF,MVV,TLC,VC |
| Mueller(2)(2013) | Switzerland | 8 | 8 | 33.5±11.7 | 41.6±17 | RMT (Carbon dioxide ventilation) | Placebo | FEV_1_,MIP,MEP,PEF,MVV,TLC,VC |
| Litchke(1)(2010) | United States | 4 | 7 | 26±7.8 | 29.1±4.3 | RMT (PowerLung ® BreatheAir trainer) | Blank | MIP,MVV |
| Litchke(2)(2010) | United States | 5 | 7 | 26.2±6.6 | 29.1±4.3 | RMT (Respiratory impedance + Concurrent Flow Resistance training) | Blank | MIP,MVV |
| Tamplin(2013) | Australia | 13 | 11 | 44±15 | 47±13 | RMT (Neuromusic Therapy) | Music appreciation and relaxation | FEV_1_,FVC,MIP,MEP, TLC,IC,VC |
| Van Houtte(1)(2008) | Belgian | 7 | 7 | 40.57±13.96 | 44.14±14.74 | RMT (Carbon dioxide ventilation) | Sham therapy | FVC,MIP,MEP,MVV |
| Van Houtte (2)(2008) | Belgian | 7 | 7 | 40.57±13.96 | 44.14±14.74 | RMT (Carbon dioxide ventilation) | Sham therapy | FVC,MIP,MEP,MVV |
| Gounden (1990) | South African | 20 | 20 | 31±7.33 | 34±10.23 | RMT (PFLEX respiratory trainer) | Conventional intervention | MEP,VC |
| Soumyashree (2020) | Indian | 15 | 12 | 29±12.6 | 34.4±13 | RMT (resistance training) | Deep breathing exercises | MIP,MEP |

Notes: *T:* Treatment group; *C:* Control group; RMT: Respiratory Muscle Training; *FEV_1_:* Forced expiratory volume in 1 second; *FVC:* Forced vital capacity; *MIP:* Maximal inspiratory pressure;*MEP:* Maximal expiratory pressure; *PEF:* Peak expiratory flow; *MVV:* Maximal voluntary ventilation; *TLC:* Total lung capacity; *IC:* Inspiratory capacity; *VC:* Vital capacity
