# Supplementary material for: Effectiveness of respiratory muscle training on pulmonary function recovery in patients with spinal cord injury: a systematic review and meta-analysis
Source: PeerJ. 2025 Nov 28;13:e20373. doi: 10.7717/peerj.20373 (PMC12667691; doi:10.7717/peerj.20373)
Supplement: Supplemental Information 3 [file peerj-13-20373-s003.docx]

| Author, year | Delivery | Thresholds | Devices | Frequency | Duration |
| --- | --- | --- | --- | --- | --- |
| Hasnakipour (2025) | Biofeedback-assisted Respiratory Motor Control Training | Not specified | Surface Electromyography Biofeedback Device (AXON 5000 Q, Canada) | 3 d per week | 5 weeks |
| Sankari (2024) | Combined oropharyngeal and respiratory muscle training | Not specified | POWERbreathe Medic Plus K-Series 3 and Expiratory Muscle Strength Trainer 150 | 30 times per day | 13 weeks |
| Luu (2023) | Progressive threshold loading for inspiratory muscles | 30% to 50% of P_I max_ | Threshold IMT device (Threshold IMT, Respironics) | 5 d per week | 6 weeks |
| Wang (2021) | High-intensity home-based RMT | 50% to 70% of MIP and MEP | Dofin Respiratory Trainer equipment | 7 d per week | 10 weeks |
| Sikka (2021) | Resistive inspiratory muscle training | Not specified | Hand-held inspiratory resistance device | 5 d per week | 4 weeks |
| Boswell-Ruys (2020) | Progressive RMT | Not specified | Threshold IMT device (Threshold IMT, Respironics) | 5 d per week | 6 weeks |
| Xi (2019) | Normocapnic hyperpnoea training | Not specified | Spirotiger (Idiag AG) | 5 d per week | 4 weeks |
| Abd El-Kader (2018) | Resistive respiratory muscle training | Not specified | Threshold positive expiratory pressure device | 5 d per week | 6 weeks |
| Kim (1)(2017) | Resistance training | Not specified | Incentive respiratory spirometer | 3 d per week | 8 weeks |
| Kim (2)(2017) | Stabilizers, abdominal traction training | Not specified | Incentive respiratory spirometer and Stabilizer | 3 d per week | 8 weeks |
| Postma (2014) | Resistive inspiratory muscle training (RIMT) | 60% of MIP at baseline | Threshold IMT device (Threshold IMT, Respironics) | 5 d per week | 8 weeks |
| West (2014) | Pressure threshold inspiratory muscle training | Not specified | POWERbreathe Plus (HaB International Ltd) | 5 d per week | 6 weeks |
| Roth (2010) | Expiratory muscle training | Not specified | Small handheld device with adjustable resistance | 5 d per week | 6 weeks |
| Liaw (2000) | Resistive inspiratory muscle training | Smallest initial resistance setting (blue, 7mm) | Diemolding Healthcare Division inspiratory muscle trainer | 7 d per week | 6 weeks |
| Loveridge(1989) | Ventilatory muscle endurance training | Not specified | Threshold load device | 5 d per week | 8 weeks |
| Derrickson(1992) | General Resistance Training | Not specified | Resistive breathing device (Threshold trainer) | 5 d per week | 7 weeks |
| Mueller(1)(2013) | Respifit S instrument | Not specified | Respifit S® (Eumedics gmbH, purkersdorf, Austria) | 4 d per week | 8 weeks |
| Mueller(2)(2013) | Carbon dioxide ventilation - IH group | Not specified | Spirotiger® (Idiag Ag, volketswil, Switzerland) | 4 d per week | 8 weeks |
| Litchke(1)(2010) | Concurrent Pressure Threshold Resistance | Not specified | PowerLung ® BreatheAir trainer | 5 d per week | 9 weeks |
| Litchke(2)(2010) | Respiratory impedance + Concurrent Flow Resistance training | Not specified | Expand-a-Lung™ device (for Concurrent Flow Resistance training) | 5 d per week | 9 weeks |
| Tamplin(2013) | Singing training involving significant respiratory demands | Not specified | Not specified | 3 d per week | 12 weeks |
| Van Houtte(1)(2008) | Normocapnic hyperpnoea training | Not specified | Normocapnic hyperpnoea device | 4 d per week | 4 weeks |
| Van Houtte (2)(2008) | Normocapnic hyperpnoea training | Not specified | Normocapnic hyperpnoea device | 4 d per week | 8 weeks |
| Gounden (1990) | Progressive resistive loading on accessory expiratory muscles | 60% of PEmax initially | PFLEX muscle trainer | 6 d per week | 8 weeks |
| Soumyashree (2020) | Inspiratory muscle training (IMT) | Not specified | Not specified | 5 d per week | 4 weeks |
