# Supplementary material for: Effectiveness of respiratory muscle training on pulmonary function recovery in patients with spinal cord injury: a systematic review and meta-analysis
Source: PeerJ. 2025 Nov 28;13:e20373. doi: 10.7717/peerj.20373 (PMC12667691; doi:10.7717/peerj.20373)
Supplement: Supplemental Information 4 [file peerj-13-20373-s004.docx]

| Study | Criteria | | | | | | | | | | | quality evaluation |
| --- | --- | --- | --- | --- | --- | --- | --- | --- | --- | --- | --- | --- |
|  | 1 | 2 | 3 | 4 | | | 5 | 6 | 7 | 8 | score (10) |  |
| Hasnakipour (2025) | ＋ | － | ＋ | － | － | － | **＋** | **＋** | ＋ | ＋ | 6 | Higher-quality |
| Sankari (2024) | ＋ | － | ＋ | ＋ | － | － | ＋ | － | ＋ | ＋ | 6 | Higher-quality |
| Luu (2023) | ＋ | － | ＋ | ＋ | ＋ | ＋ | ＋ | － | ＋ | ＋ | 8 | Higher-quality |
| Wang (2021) | ＋ | － | ＋ | ＋ | ＋ | ＋ | ＋ | － | ＋ | ＋ | 8 | Higher-quality |
| Sikka (2021) | ＋ | － | ＋ | － | － | － | ＋ | － | ＋ | ＋ | 5 | Average-quality |
| Boswell-Ruys (2020) | ＋ | ＋ | ＋ | ＋ | ＋ | ＋ | ＋ | ＋ | ＋ | ＋ | 10 | High-quality |
| Xi (2019) | ＋ | － | ＋ | － | ＋ | － | ＋ | － | ＋ | ＋ | 6 | Higher-quality |
| Abd El-Kader (2018) | ＋ | － | ＋ | － | － | － | ＋ | － | ＋ | ＋ | 5 | Average-quality |
| Kim (2017) | ＋ | － | ＋ | ＋ | ＋ | ＋ | ＋ | － | ＋ | ＋ | 8 | Higher-quality |
| Postma (2014) | ＋ | ＋ | ＋ | ＋ | － | ＋ | ＋ | － | ＋ | ＋ | 8 | Higher-quality |
| West (2014) | ＋ | ＋ | ＋ | ＋ | － | ＋ | ＋ | － | ＋ | ＋ | 8 | Higher-quality |
| Roth (2010) | ＋ | － | ＋ | － | － | ＋ | ＋ | － | ＋ | ＋ | 6 | Higher-quality |
| Liaw (2000) | ＋ | － | ＋ | － | － | ＋ | ＋ | － | ＋ | ＋ | 6 | Higher-quality |
| Loveridge (1989) | ＋ | － | ＋ | ＋ | － | － | ＋ | － | ＋ | ＋ | 6 | Higher-quality |
| Derrickson (1992) | ＋ | － | ＋ | － | － | ＋ | ＋ | － | ＋ | ＋ | 6 | Higher-quality |
| Mueller (2013) | ＋ | － | ＋ | ＋ | － | ＋ | ＋ | － | ＋ | ＋ | 7 | Higher-quality |
| Litchke (2010) | ＋ | － | ＋ | ＋ | － | － | ＋ | － | ＋ | ＋ | 6 | Higher-quality |
| Tamplin (2013) | ＋ | ＋ | ＋ | ＋ | － | ＋ | ＋ | － | ＋ | ＋ | 8 | Higher-quality |
| Van Houtte (2008) | ＋ | － | ＋ | ＋ | － | － | ＋ | － | ＋ | ＋ | 6 | Higher-quality |
| Gounden (1990) | ＋ | － | ＋ | － | － | － | ＋ | － | ＋ | ＋ | 5 | Average-quality |
| Soumyashree(2020) | ＋ | ＋ | ＋ | － | ＋ | － | ＋ | － | ＋ | ＋ | 7 | Higher-quality |

Item score: “+”= 1 point for meeting criteria,“－”= 0 points for not meeting criteria; Criteria:1 = Random allocation, 2 = Concealed allocation, 3 = Baseline comparability,4 = Blind(subjects, therapists,assessors) ,5 = Follow-up＞85%,6 = Intention to Treat Analysis, 7 = Between-group comparisons, 8 = Pointestimates and variability.
